# NAD^+^ ameliorates endotoxin‐induced acute kidney injury in a sirtuin1–dependent manner via GSK‐3β/Nrf2 signalling pathway

**DOI:** 10.1111/jcmm.17222

**Published:** 2022-02-09

**Authors:** Simeng He, Qiaoying Gao, Xiaoyang Wu, Jia Shi, Yuan Zhang, Jing Yang, Xiangyun Li, Shihan Du, Yanfang Zhang, Jianbo Yu

**Affiliations:** ^1^ School of Medicine Nankai University Tianjin China; ^2^ Tianjin key Laboratory of Acute Abdomen Disease Associated Organ Injury and ITCWM Repair Institute of Acute Abdominal Diseases of Integrated Traditional Chinese and Western Medicine Tianjin Nankai Hospital Tianjin China; ^3^ Department of Anesthesiology and Critical Care Medicine Tianjin Nankai Hospital Tianjin Medical University Tianjin China

**Keywords:** acute kidney injury, NAD^+^, renal tubular injury, sepsis, sirtuins

## Abstract

Acute kidney injury (AKI) is a substantial worldwide public health concern with no specific and effective therapies in clinic. NAD^+^ is a pivotal determinant of cellular energy metabolism involved in the progression of AKI; however, its mechanism in kidney injury remains poorly understood. Sirtuin 1 (SIRT1) is an NAD^+^‐dependent deacetylase associated with renal protection and acute stress resistance. In this study, we have investigated the role of NAD^+^ in AKI and the potential mechanism(s) involved in its renoprotective effect. NAD^+^ was notably decreased and negatively correlated with kidney dysfunction in AKI, restoring NAD^+^ with NMN significantly ameliorates LPS‐induced oxidative stress and apoptosis and attenuates renal damage. We also found that the protection of NAD^+^ is associated with SIRT1 expressions and performs in a SIRT1‐dependent manner. Inhibition of SIRT1 blunted the protective effect of NAD^+^ and up‐regulated the activity of glycogen synthase kinase‐3β (GSK‐3β) that was concomitant with mitigated Nrf2 nuclear accumulation, thereby exacerbates AKI. These findings suggest that NAD^+^/SIRT1/GSK‐3β/Nrf2 axis is an important mechanism that can protect against AKI which might be a potential therapeutic target for the treatment of AKI.

## 1 INTRODUCTION

Sepsis‐induced acute kidney injury (AKI) is a frequent complication seen in hospitalized and critically ill patients and is associated with an increased length of hospital stay, development of chronic comorbidities and extremely high mortality.^1^, ^2^ Since the pandemic of coronavirus disease 2019 (COVID‐19), the kidney is more vulnerable to targeted attacks because the binding site for SARS‐CoV‐2 (angiotensin‐converting enzyme 2) is highly expressed in proximal tubule cells and podocytes.^3^, ^4^ Although the growing healthcare burden of the AKI has progressively increased worldwide, there is currently no satisfactory therapy for preventing and treating AKI,^5^, ^6^, ^7^ also an urgent need to discover new renoprotective therapies for improving clinical outcomes in patients with AKI.

NAD^+^ (nicotinamide adenine dinucleotide) is a ubiquitous hydride acceptor that plays an essential role in energy metabolism and adaptive stress responses.^8^, ^9^ The kidney is both among the highest mitochondria and NAD^+^ containing organs, yet is also highly susceptible to NAD^+^ depletion.^10^, ^11^ Oxidative stresses and mitochondrial damage are drivers of sepsis‐induced AKI, followed by cellular NAD^+^ depletion and NAD^+^ synthesis reduction.^10^, ^12^, ^13^ Accumulation studies suggest that elevating intracellular NAD^+^ levels, including regulation of critical enzymes in NAD^+^ biosynthesis and exogenous supplementation of its precursors, are potential therapeutic strategies for renal diseases in model organisms ranging from mice to humans.^14^, ^15^, ^16^, ^17^ However, the mechanisms by which effectiveness remains incompletely understood.

Sirtuin 1 (SIRT1) is an NAD^+^‐consuming deacetylase associated with cytoprotective effects exerted by the inhibition of inflammasome activation, deacetylation of downstream proteins (such as p53, nuclear factor‐κB (NF‐κB), forkhead box protein (Fox) and peroxisome proliferator‐activated receptor‐γ co‐activator‐1α (PGC‐1α)) and induction catalase activity, which lead to anti‐inflammatory, anti‐apoptotic and antioxidant effects.^18^, ^19^, ^20^ Studies have demonstrated that SIRT1 activation attenuates renal oxidative stress and apoptosis and improves the decline of renal functions.^21^, ^22^, ^23^ Therefore, SIRT1 might be a therapeutic agent for the treatment of renal disorders. It has been well documented that nicotinamide mononucleotide (NMN), an NAD^+^ precursor, rescues age‐related susceptibility to cisplatin‐induced AKI depended on SIRT1 which contributes to the resistance of aging.^19^, ^24^ Thus, it seemed reasonable to hypothesize that the effect of NAD^+^ on LPS‐induced AKI is associated with SIRT1.

In this study, we examined that the role of NAD^+^/SIRT1 in sepsis‐induced AKI. Our study suggests that NAD^+^ supplementation contributed to rescued the renal damage and its deficits increased vulnerability to sepsis‐induced AKI, which performed in a SIRT1‐dependent manner. Furthermore, the mechanism by which NAD^+^/SIRT1 alleviates the sepsis‐induced AKI involves the regulation of glycogen synthase kinase‐3β (GSK‐3β) / nuclear factor erythroid 2‐related factor 2 (Nrf2) signalling pathway. The study, therefore, sheds light on the mechanisms underlying the renoprotective capacity of NAD^+^ and identifies NAD^+^/SIRT1/GSK‐3β/Nrf2 pathway as a potential therapeutic target, which might be used to prevent or ameliorate sepsis‐induced AKI.

## 2 MATERIALS AND METHODS

### 2.1 Animals

Wild‐type C57BL/6 mice (aged 6~8 weeks, weighting 20~22 g, male) were purchased from Vital River. NMNAT1 (the critical enzyme for NAD^+^ salvage biosynthetic pathway) conditional‐knockout (^−/−^) mice on a C57BL/6 background (NMNAT1^flox^R26^CreERT2^) were purchased by Beijing Biocytogen Co., Ltd, in which the NMNAT1 gene was induced by tamoxifen (Sigma) dissolved in corn oil at a concentration of 10 mg/ml, which injected intraperitoneally once a day for a total of five consecutive days and waited for one week. All mice were housed at 25°C with 12 h light and dark photocycle, and they were free access to food and water ad libitum throughout the study period. The study was performed in accordance with the legislation of laboratory animals and approved by the Animal Care and Use Committee of the Tianjin Nankai Hospital (Approval No. NKYY‐DWLL‐2019–012, Tianjin, China).

### 2.2 Experimental protocol

To evaluate the effect of NAD^+^ on LPS‐induced AKI, wild‐type and NMNAT1^−/−^ mice were assigned to four groups, respectively: control (CON); LPS‐treated mice (LPS); LPS plus NMN‐treated mice (LPS + NMN) and NMN‐treated mice (NMN). All mice were anaesthetized with 2%~3% isoflurane (R510‐22, RWD Life Science) inhalation for induction and 1.5% isoflurane for maintenance. Wild‐type and NMNAT1^−/−^ mice were used in the LPS‐induced AKI model by caudal vein injection of LPS (E.coli‐L2630, Sigma) 15 mg/kg diluted in 2 ml saline as described previously.^25^ In addition, the mice were intraperitoneally injected with 500 mg/kg NMN (M3501, Sigma, USA) for 7 consecutive days prior to LPS treatment. To explore the effect of SIRT1 on LPS‐induced AKI and the involved GSK‐3β/Nrf2 signalling pathway, wild‐type C57BL/6 mice were divided into four groups: control (CON); LPS‐treated mice (LPS); LPS plus SIRT1 selective agonist (SRT‐1720)‐treated mice (LPS + SRT‐1720) and LPS plus SIRT1 specific inhibitor‐treated mice (LPS + EX‐527). Furthermore, to determine the effect of NAD^+^ on AKI depends on SIRT1, LPS plus NMN‐treated mice with or without EX‐527: LPS + NMN group; LPS + NMN + EX‐527 group, mice were injected with SIRT1 agonist SRT‐1720 (30 mg/kg, SC0267, Beyotime) or SIRT1 inhibitor EX‐527 (5 mg/kg, SC0281, Beyotime) intraperitoneally before LPS injection as described before.^26^, ^27^

Twelve hours after LPS treatment, all the mice were sacrificed under deep anaesthesia via cervical dislocation. Blood samples were collected through cardiac puncture and centrifuged 3000 g for 10 min at 4℃, and the isolated serum was stored at −80℃. Kidney tissues were fixed in 4% paraformaldehyde for pathological analyses, and remaining kidney tissues were stored at −80℃ for further analyses.

### 2.3 Haematoxylin and eosin staining for renal pathology

To evaluate renal histopathology changes, kidney tissue was immediately collected and fixed overnight with 4% paraformaldehyde, embedded in paraffin, and subsequently sectioned at a 4 μm thickness for further haematoxylin and eosin staining according to standard procedures. A minimum of 10 fields on each slide were examined and scored for pathological injury by a pathologist in a blinded fashion. The tubular injury score from 0 to 4 was given: 0, normal; 1, mild injury (5%~25% of tubules were damaged); 2, moderate injury (25%~50% of tubules were damaged); 3, severe injury (50%~70% tubules were damaged and 4, almost all tubules in the field were damaged), which was based on a standard previously reported.^28^, ^29^ Images were acquired with Leica DM4000B microscopy (Leica).

### 2.4 Assessment of kidney function

Blood urea nitrogen (BUN) and serum creatinine (Scr) were assayed as indicators of renal functions, and kidney injury molecule‐1 (KIM‐1) and neutrophil‐gelatinase‐associated lipocalin (NGAL) have been identified as renal injury biomarkers to indicate AKI.^30^ The serum collected was measured for BUN and Scr levels using AutoAnalyzer (Roche Diagnostics) and KIM‐1 and NGAL levels using enzyme‐linked immunosorbent assay (enzyme‐linked Biotechnology Co., Ltd).

### 2.5 NAD^+^ measurement

NAD^+^ concentrations from the kidney were measured with an NAD/NADH Assay Kit (KA1657, Abnova) according to the previous report.^31^ The assay is based on the lactate dehydrogenase cycling reaction, in which the formed NADH reduces a formazan (MTT) reagent. Frozen kidney tissue (20 mg) was prepared for homogenizing samples and transferred into a 1.5 ml Eppendorf tube with either 100 μl NAD or NADH extraction buffer for NAD and NADH determination respectively. The extracts were heated at 60℃ for 5 min, followed by 20 μl assay buffer and 100μl the opposite extraction buffer to neutralize the solutions. After centrifugation at 14,000 *g* for 5 min, the supernatant was obtained to determine the concentrations of NAD and NADH. The NAD^+^ content was calculated as the total NAD content minus the NADH content.

### 2.6 Measurement of mitochondrial DNA content

Mitochondrial DNA (mtDNA) is tightly associated with mitochondrial enzyme activity and ATP production and is therefore indicated as a biomarker of mitochondrial function.^32^ The mtDNA concentrations were measured by RT‐PCR as previously described.^33^ Briefly, total RNA from kidneys was isolated by a RNeasy Mini Kit (Qiagen, Hilden, Germany) according to the manufacturer's instructions. Isolated RNA from kidneys was converted into cDNA using a cDNA synthesis kit (TaKaRa) under the following conditions: 37°C for 15 min, 85°C for 5 s, followed by 4°C for 10 min (T100 Thermal Cycler, Bio‐Rad). Then, RT‐PCR was performed using the Applied Biosystems 7500 real‐time PCR system in a total volume of 20 μl, including TB Green Premix Ex Taq II 10 μl, forward primer 0.8 μl, reverse primer 0.8 μl, ROX reference dye 0.4 μl, cDNA 2 μl and DNase/RNase‐free dH_2_O 6 μl in the following conditions: pre‐incubation at 95℃ for 30 s, 40 cycles of denaturation at 95℃ for 5 s, annealing and extension at 60℃ for 34 s. The primers of mtDNA were as follows: forward 5′‐CCCAGCTACTACCATCATTCAAGT‐3′, reverse 5′‐GATGGTTTGGGAGATTGGTTGATG‐3′; the primers of nuclear‐encoded internal were: forward 5′‐ GCCAGCCTGACCCATAGCCATAATAT‐3′ and reverse 5′‐GAGAGATTTTATGGGTGTAATGCGG‐3′. Calculations of the mtDNA contents were based on the $2^{‐\Delta \Delta C_{t}}$ method.

### 2.7 Detection of mitochondrial ROS

A mitochondria isolation kit (Thermo Scientific) was used for mitochondria fraction preparation. Mitochondrial ROS was measured by using 2’,7’‐dichlorofluorescein diacetate (DCFH‐DA) probe (Solarbio) for 30 min at room temperature, then, washed three times with PBS and collected in a glass slide containing PBS. The fluorescence signal was measured, respectively, at excitation and emission wavelength of 488 nm and 520 nm by a microplate reader. The fluorescent images were assessed using Image Pro‐Plus software and the results for CON (WT) group were expressed as a relative reference.

### 2.8 Oxygen consumption rate

The mitochondrial respiration was examined by analysing the mitochondrial oxygen consumption rate (OCR) with an XFe96 extracellular flux analyzer (Agilent Technologies). Briefly, the isolated mitochondrial pellet was gently resuspended and was incubated in a CO_2_‐free environment for 1 h and OCR was determined every 3 min over 90 min. OCR was measured in basal conditions: 20 mM glucose, 1 μmol/L oligomycin (ATP synthase inhibitor), 0.125 μmol/L FCCP (mitochondrial respiration uncoupler) and 1 μmol/L retenone/antimycin A (complex I and III inhibitors respectively).^34^

### 2.9 Electron microscopy

Tissues of the kidneys were fixed in 2.5% glutaraldehyde in 0.1 M phosphate buffer (PH 7.4) for 1 h at 4℃. Specimens were post‐fixed with 1% osmium tetroxide in 0.1 M cacodylate buffer for 2 h at 4℃, after which they were then dehydrated in graded ethanol and then embedded in acetone and sectioned approximately 70nm. Ultrathin sections were stained with 3% uranyl acetate and lead citrate. Observations were performed on a JEM‐1230 transmission electron microscope (JEOL, Japan) at 80 kV.

### 2.10 TUNEL assay

The cell death was evaluated by a TdT‐mediated dUTP nick‐end‐labelling (TUNEL) in situ cell death detection kit (KGA7072, keyGEN BioTECH) that followed the manufacturer's protocols. Detection of the apoptotic cells exhibiting green fluorescence was quantified by Leica DM4000B microscopy and represented as percentages of TUNEL‐positive cells out of total cells in the fields.

### 2.11 Immunostaining

Preparation of paraffin tissue sections was performed as described above. The immunohistochemistry (IHC) analysis was performed according to the following procedures. Briefly, renal sections were deparaffinized, and the slides were in modified Citrate Antigen Retrieval Solution (P0083, Beyotime, China) for 1 min and then cooled for 20 min. After incubation in 3% hydrogen peroxide for 15 min, sections were blocked with 10% normal goat serum at 37°C for 60 min, and then incubated overnight 4℃ with rabbit monoclonal primary antibodies against inducible nitric oxide synthase (iNOS, 1:100, ab115819), GSK‐3β (1:200; ab93926) and Nrf2 (1:200; ab137550). The sections were washed in TBST three times for 5 min, then incubated for 60 min at room temperature with horseradish peroxidase‐conjugated anti‐rabbit secondary antibody followed by a DAB kit (ZLI‐9018, zsbio). For immunofluorescence (IF), the sections were incubated overnight with the primary antibody SIRT1 (1:30, ab189494) at 4°C. After washing, slices were incubated with secondary antibody fluorescein isothiocyanate (FITC)‐conjugated IgG at 37°C for 30 min. All image were photographed using a Leica DM4000B microscopy and was calculated using the ImageJ soft.

### 2.12 Quantitative RT‐PCR

Total RNA from kidneys was extracted using a RNeasy Mini Kit (Qiagen) in accordance with the manufacturer's manuals. Isolated RNA (500 ng) was converted into cDNA using Prime‐Script RT reagent Kit (TaKaRa Biotechnology Co., Ltd.) under the conditions: 37℃ for 15 min, 85℃ for 5 s and 4℃ for 10 min using the T100 Thermal Cycler (Bio‐Rad). RT‐PCR was performed using the 7500 real‐time PCR system (Applied Biosystems, USA) with the specific primers of SIRT1: forward 5′‐CGGCTACCGAGGTCCATATAC‐3′; reverse 5′‐CAGCTCAGGTGGAGGAATTGT‐3′. β‐actin: forward 5′‐GAGGCCCAGAGCAAGAGAGGT‐3′ and reverse 5′‐ TTCACGGTTGGCCTTAGGGTT‐3′. PCR conditions were as follows: pre‐degeneration was performed at 95 ℃ for 30 s, 40 cycles of denaturation at 95℃ for 5 s, annealing and extension at 60℃ for 34 s. The relative mRNA levels for the specific genes were normalized to GAPDH mRNA and calculated by the $2^{‐\Delta \Delta C_{t}}$ method.

### 2.13 Western blot analysis

The Nuclear and Cytoplasmic Protein Extraction Kit (Beyotime) was used for cytoplasmic (for total Nrf2) and nuclear proteins (for nuclear Nrf2 and Histone H3) extraction. Total proteins were extracted from kidneys using a total protein isolation kit (Thermo Fischer Scientific) and its concentrations were determined by the BCA protein assay kit (Sigma). Equal amounts of protein were separated on 10% SDS‐PAGE gel and then were transferred to a PVDF membrane (0.2 µM, Bio‐Rad, USA). After blocking with 5% skim milk in TBST for 3h and incubated overnight at 4℃ with primary antibody against SIRT1(1:1000, ab189494), Bax (1:1000, CST5023), Bcl‐2 (1:1000, CST3498), cleaved‐caspase3 (1:500, CST9664s), anti‐phospho‐Ser9‐GSK‐3β (1:1000, CST 9336), anti‐GSK‐3β (1:1000, ab93926), anti‐Nrf2 (1:1000, ab137550), anti‐histone H3 (1:1000, CST4499) and β‐actin (1:1000, AF7018). Subsequently, the membranes were washed with TBST 5 times (5 min for each time) and then incubated with HRP‐labelled secondary antibodies (1:2000, S0001) for 1 h at room temperature. The blots were visualized using an enhanced chemiluminescence Western blot detection kit (170–5070, Bio‐Rad), and the relative expression of target proteins was quantified by the Image‐Analysis system.

### 2.14 Statistical analysis

All values were expressed as mean ±*SD* and Student's *t* test or one‐way analysis of variance (ANOVA) followed by Bonferroni post hoc test were conducted for comparisons among groups. Statistical analyses were carried out using GraphPad Prism 8.3.0 (GraphPad Software Inc.), and *p* values of ≤0.05 were accepted as statistically significant.

## 3 RESULTS

### 3.1 NAD^+^ supplementation ameliorates LPS‐induced AKI whereas its deficiency aggravates kidney injury

**FIGURE 1:**
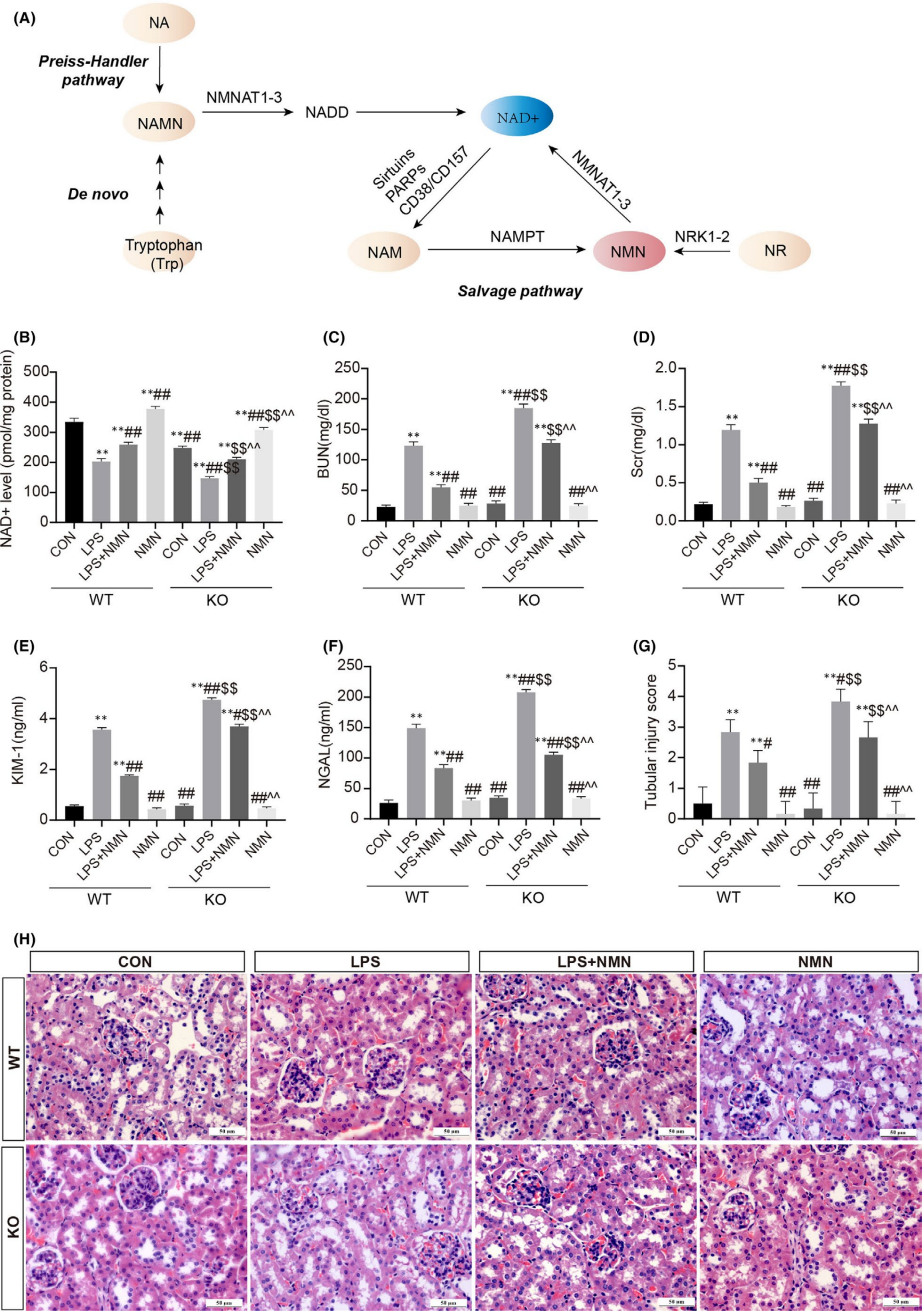
NAD^+^ plays a critical role in LPS‐induced kidney damage. (A) NAD^+^ metabolism. (B) NAD^+^ levels in kidney cortexes of wild‐type (WT) and NMNAT1^−/−^ (KO) mice under different conditions. The levels of BUN (C) and serum creatinine (D) in the indicated groups. Hallmarks of renal damage evaluated by kidney injury molecule 1 (KIM‐1) (E) and neutrophil gelatinase‐associated lipocalin (NGAL) (F) were analysed using enzyme‐linked immunosorbent assay. Kidney specimens from each group were evaluated using haematoxylin and eosin (H&E) staining (scale bars, 50 μm) (H), following by tubular injury scores assessment (G). Each result was replicated in three independent experiments, and all values are the mean ±*SD* (*n* = 3). *Significance compared with CON (WT) group (*p < 0.05, **p < 0.01). ^#^Significance compared with LPS (WT) group (^#^ *p *< 0.05, ^##^ *p *< 0.01). ^$^Significance compared with CON (KO) group (^$^ *p *< 0.05, ^$$^ *p *< 0.01). ^^^Significance compared with LPS (KO) group (^^^ *p *< 0.05, ^^^^ *p *< 0.01)

Substantial evidence indicates that NAD^+^ is a ubiquitous coenzyme that functions as a guardian against oxidative stress.^35^ NMNAT1 has been identified as the highest catalytic properties in NAD^+^ biosynthesis, and NMN supplementation can increase NAD^+^ availability via the NAD^+^ salvage pathway in mice^17^, ^36^ (Figure 1A); hence knockout *NMNAT1* conditional and exogenous NMN supplementation were designed to emulate altered NAD^+^.

To elucidate the role of NAD^+^ in LPS‐induced AKI, wild‐type (WT) and NMNAT1‐deficient (KO) mice were subjected to LPS treatment to induce AKI. An apparent reduction in NAD^+^ concentrations was found in kidneys with AKI of WT mice relative to that in controls. The levels of NAD^+^ in the kidneys of NMNAT1^−/−^ mice were lower than those in the kidneys of WT mice under the same conditions, and NMN treatment restored NAD^+^ contents significantly (Figure 1B). Next, we detected the renal functional markers blood urea nitrogen (BUN) and serum creatinine (SCr), the expressions of the tubular injury markers kidney injury molecule 1 (KIM‐1) and neutrophil gelatinase–associated lipocalin (NGAL) and the assessment of histopathologic changes to quantify the renal injury (Figure 1C‐H). The levels of BUN, SCr, KIM‐1 and NGAL were markedly elevated and tubular histopathologic changes appeared both in WT and NMNAT1^−/−^ mice subjected to LPS, conforming to the characteristics of AKI (Figure 1C‐H). However, NMNAT1^−/−^ mice subjected to LPS exhibited exacerbated tubular injury and worsening of renal function as compared with wild‐type mice, indicating that NAD^+^ depletion exacerbated the extent of AKI (Figure 1C‐H). Exogenous supplementation of the NAD^+^ precursors NMN significantly reduced the levels of all four markers and attenuated the tubular necrosis, suggesting that restoring NAD^+^ ameliorated LPS‐induced renal dysfunction and structural damage (Figure 1C‐H). The pathological semi‐quantitative evaluation by the percentage of damaged tubules in a blind manner further confirmed that NAD^+^ ameliorates LPS‐induced renal dysfunction while its deficiency aggravates the injury.

### 3.2 NAD^+^ attenuates LPS‐driven apoptosis in renal tissues.

**FIGURE 2:**
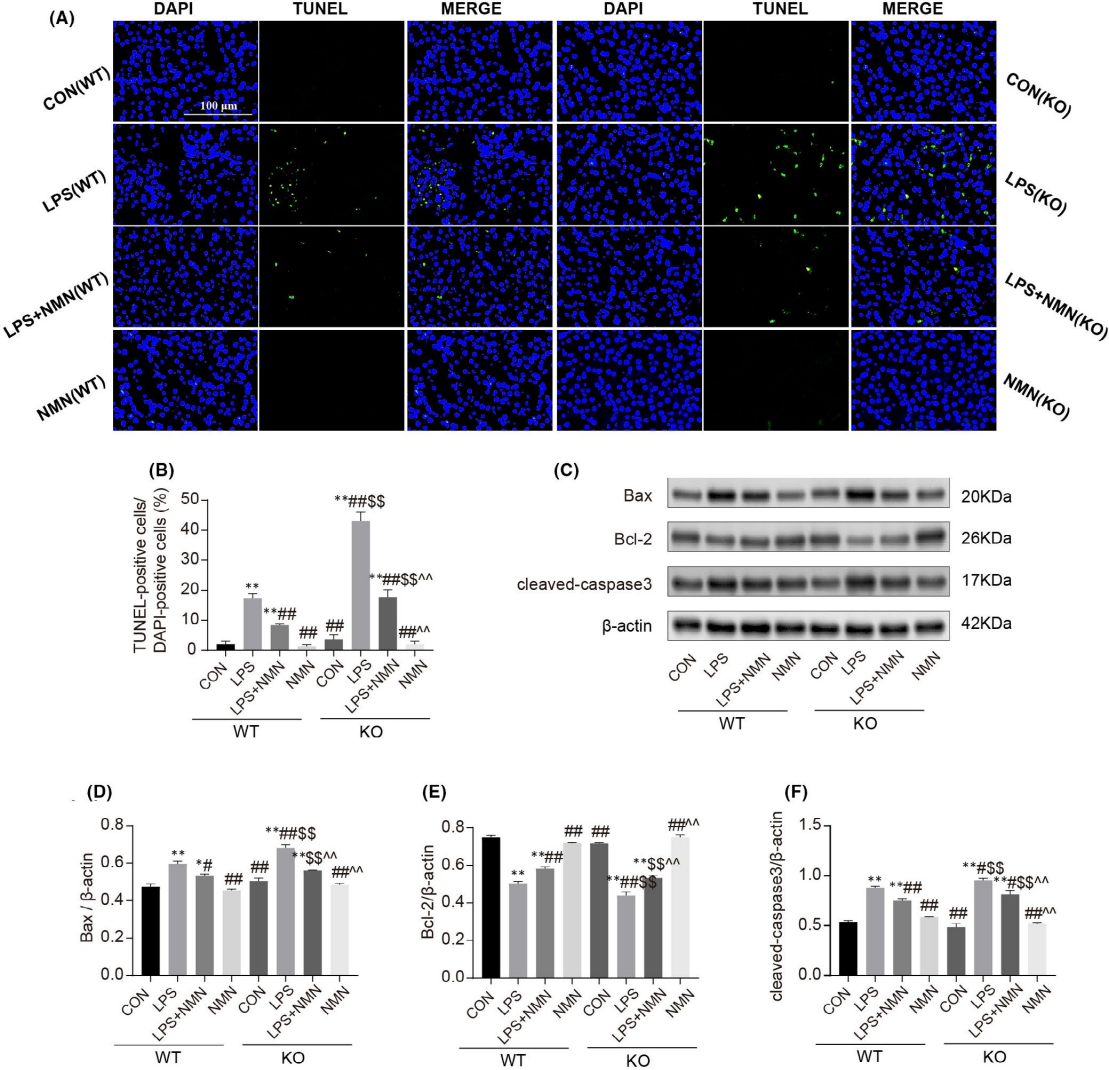
Effect of NAD^+^ on LPS‐induced apoptosis in renal tissues. (A‐B) Representative images of the TUNEL staining on kidneys (scale bars, 100 μm) and counted percentages of terminal deoxynucleotidyltransferase‐mediated dUTP nick end‐labelling (TUNEL)‐positive cells. (C‐F) Representative immunoblotting analysis of Bax, Bcl‐2 and cleaved‐caspase3. Each result was replicated in three independent experiments, and all values are the mean ±*SD* (*n *= 3). *Significance compared with CON (WT) group (*p < 0.05, **p < 0.01). ^#^Significance compared with LPS (WT) group (^#^ *p *< 0.05, ^##^ *p *< 0.01). ^$^Significance compared with CON (KO) group (^$^ *p *< 0.05, ^$$^ *p *< 0.01). ^^^Significance compared with LPS (KO) group (^^^ *p *< 0.05, ^^^^ *p *< 0.01)

Apoptosis is well‐described as a feature of LPS‐induced AKI.^37^ To investigate the effects of NAD^+^ on LPS‐triggered renal apoptosis, we performed terminal deoxynucleotidyltransferase‐mediated dUTP nick‐end‐labelling (TUNEL) staining on different conditions. We found more TUNEL‐positive cells in LPS‐treated wild‐type and NMNAT1^−/−^ mice relative to controls, respectively, and fewer TUNEL‐positive cells were shown in NMN supplementation (Figure 2A‐B). Three apoptotic markers independently confirmed these results, the expressions of Bax and cleaved‐caspase3 increased after treatment with LPS both in wild‐type and NMNAT1^−/−^ mice, and the increases were diminished by the administration of NMN, while the expression of Bcl‐2 showed the opposite trend (Figure 2C‐F). These results suggest that NAD^+^ protects the kidney from LPS‐induced renal apoptosis.

### 3.3 NAD^+^ protected against mitochondrial oxidative damage and improved mitochondrial morphology in LPS‐induced AKI

**FIGURE 3:**
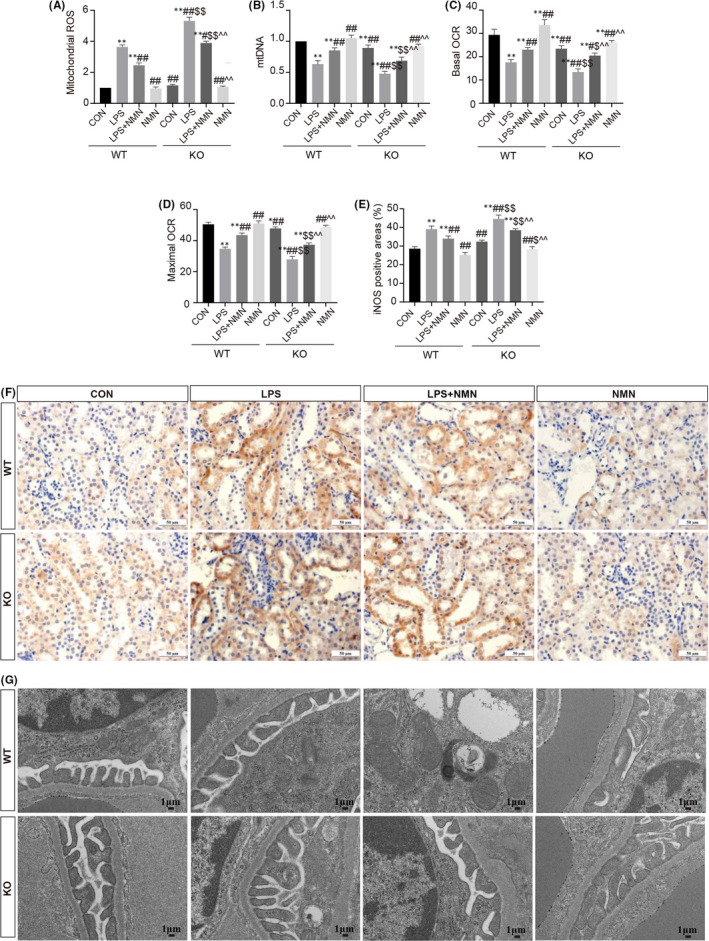
NAD^+^ protects against LPS‐induced mitochondrial dysfunctions and oxidative stress. (A) Levels of mitochondrial ROS were determined using a commercial kit in each group. (B) qRT‐PCR analysis for mtDNA copy numbers in kidneys. (C‐D) Basal OCR and maximal OCR represented as the basal mitochondrial OXPHOS activity and maximal respiratory capacity respectively. (E‐F) Kidney specimens in each group were processed for immunohistochemistry staining for iNOS (scale bars, 50 μm) and quantitative analysis for positive areas. (G) Kidney tissue was processed for electron micrographs of renal mitochondria (scale bars, 1 μm). Each result was replicated in three independent experiments and all values are the mean ±*SD* (*n* = 3). *Significance compared with CON (WT) group (*p<0.05, **p < 0.01). ^#^Significance compared with LPS (WT) group (^#^ *p *< 0.05, ^##^ *p *< 0.01). ^$^Significance compared with CON (KO) group (^$^ *p *< 0.05, ^$$^ *p *< 0.01). ^^^Significance compared with LPS (KO) group (^^^ *p *< 0.05, ^^^^ *p *< 0.01)

Oxidative stress is confirmed as a major hallmark of LPS‐induced AKI and is characterized by the accumulation of reactive oxygen species (ROS), impaired antioxidant capability as well as mitochondrial dysfunction.^38^, ^39^ As shown in Figure 3, exposure to LPS both in WT and KO mice showed a striking increase in the levels of mitochondrial ROS and inducible nitric oxide synthase (iNOS) contents while a marked reduction of mtDNA and OCR, which were significantly suppressed by pre‐processing with NMN. Correspondingly, the normal nuclear and mitochondrial morphology were shown in control groups, whereas LPS caused mitochondria vacuolization and nuclear karyopyknosis, and NMN pretreatment dramatically improved the ultrastructural damage. In addition, compared with CON (WT) group, mtDNA and OCR in CON (KO) group were significantly reduced, which might be contributed to the critical role of NAD^+^ in cellular energy metabolism. Moreover, those functional and morphological changes were exacerbated in LPS‐treated NMNAT1^−/−^ mice relative to LPS‐treated WT mice. Collectively, these results demonstrated that NAD^+^ preserves mitochondrial function and morphology that are important in AKI.

### 3.4 The protection of NAD^+^ is associated with SIRT1 expression

**FIGURE 4:**
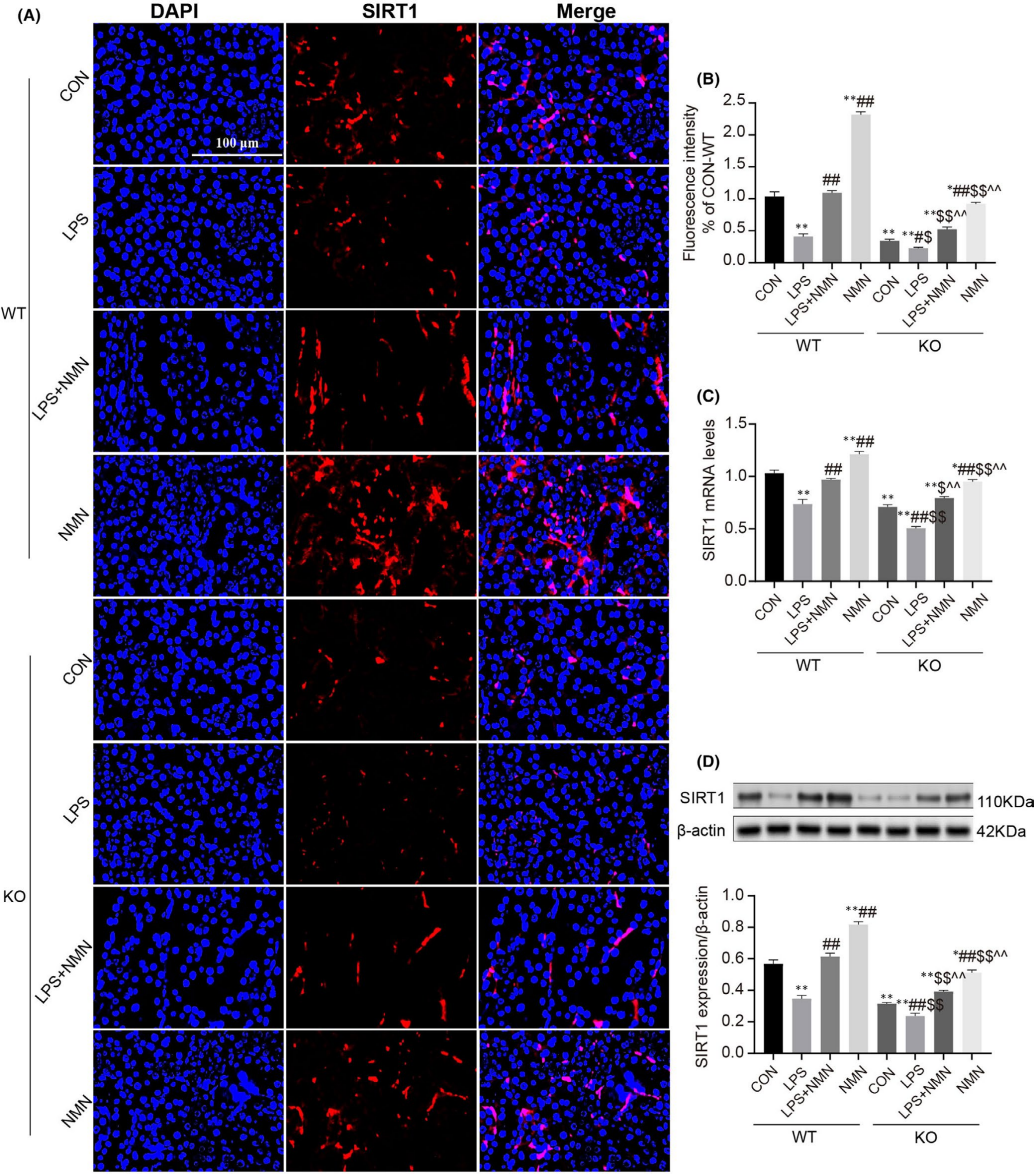
Effect of NAD+on LPS‐induced AKI is associated with SIRT1. (A‐B) Immunofluorescence intensity of renal SIRT1 (red) in different groups (scale bars, 100 μm). (C) The relative mRNA expression of SIRT1 in kidney cortexes. (D) Representative Western blots analysis and quantitation of band density of SIRT1 expression in different groups. Each result was replicated in three independent experiments and all values are the mean ±*SD* (*n* = 3). *Significance compared with CON (WT) group (*p < 0.05, **p < 0.01). ^#^Significance compared with LPS (WT) group (^#^ *p *< 0.05, ^##^ *p *< 0.01). ^$^Significance compared with CON (KO) group (^$^ *p *< 0.05, ^$$^ *p *< 0.01). ^^^Significance compared with LPS (KO) group (^^^ *p *< 0.05, ^^^^ *p *< 0.01)

SIRT1 is a highly conserved NAD^+^‐dependent deacetylase known to have protective effects against a variety of renal diseases.^22^, ^23^, ^40^ To elucidate whether the protection of NAD^+^ in LPS‐induced AKI is positively associated with the expression of SIRT1, we firstly observed the changes of SIRT1 in different concentrations regimes of NAD^+^. As shown in Figure 4, an apparent reduction of SIRT1 expression was found in kidneys from NMNAT1^−/−^ mice relative to wild‐type. LPS treatment remarkably decreased the mRNA and protein expressions of SIRT1 both in wild‐type and NMNAT1^−/−^ mice, the results were consistent with the previous study.^41^, ^42^ Additionally, the expressions of SIRT1 were restored by NMN supplementation, confirming that SIRT1 levels were responsive to intracellular NAD^+^ concentration. Briefly, the tendency of SIRT1 was entirely similar for NAD^+^, and it was reasonable to believe that these two events interact which indicated the protection of NAD^+^ is correlated with SIRT1 expression.

### 3.5 SIRT1 protects against LPS‐induced renal dysfunction and apoptosis

**FIGURE 5:**
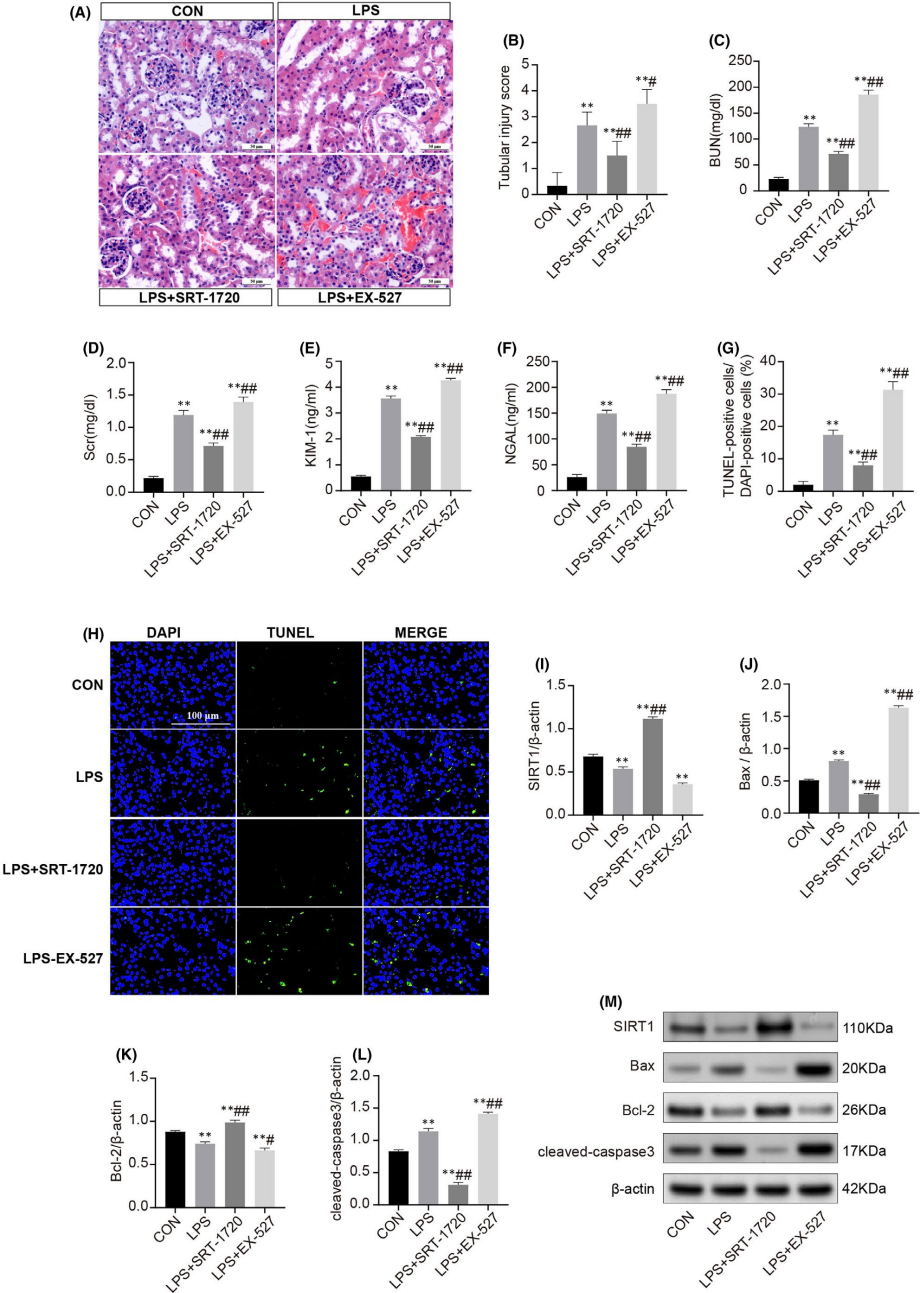
SIRT1 alleviates acute kidney injury and apoptosis. (A) Representative haematoxylin and eosin‐stained (H&E) kidney sections of C57BL/6 mice which underwent LPS administration and were treated with SRT‐1720 or EX‐527 (scale bars, 50μm). (B) Histopathological score of H&E staining of renal tissue. Quantitative analysis of BUN (C), Scr (D), KIM‐1 (E) and NGAL (F) in the different groups. (G‐H) Representative images of the TUNEL staining on kidneys (scale bars, 100 μm) and statistical percentages of TUNEL‐positive cells. (I‐M) Representative Western blots and statistical results of the protein expressions of SIRT1, Bax, Bcl‐2 and cleaved‐caspase3. Each result was replicated in three independent experiments, and all values are the mean ±*SD* (*n* = 3). *Significance compared with CON group (*p < 0.05, **p < 0.01). #Significance compared with LPS group (^#^ *p *< 0.05, ^##^ *p *< 0.01)

To directly observe the role of SIRT1 in the LPS‐driven AKI model, we used the SIRT1 selective activator (SRT‐1720) and specific inhibitor (EX‐527) for LPS‐treated mice respectively. The SIRT1 activator SRT‐1720 significantly reduced the BUN, Scr, KIM‐1, NGAL levels in LPS‐induced AKI, and its effect on kidneys was also supported by histological assessment (Figure 5A‐F). At the same time, we found fewer TUNEL‐positive cells in LPS+SRT‐1720 mice kidneys compared with the LPS group, the pro‐apoptotic proteins Bax and cleaved‐caspase3 were down‐regulated markedly while anti‐apoptotic protein Bcl‐2 was up‐regulated (Figure 5G‐M). Conversely, the inhibitor of SIRT1 aggravated the kidney injury was shown as increase of biomarkers of acute renal injury (KIM‐1, NGAL) and renal failure (BUN, Scr) with more severe renal histopathologic impairment (Figure 5A‐F). Consistently, TUNEL‐positive cells and apoptotic‐related proteins Bax and cleaved‐caspase3 were significantly increased while the expression of Bcl‐2 had a significant reduction. These results suggest that SIRT1 alleviates the LPS‐induced AKI and decreases apoptosis.

### 3.6 The effect of NAD^+^ on the kidney depends on SIRT1

**FIGURE 6:**
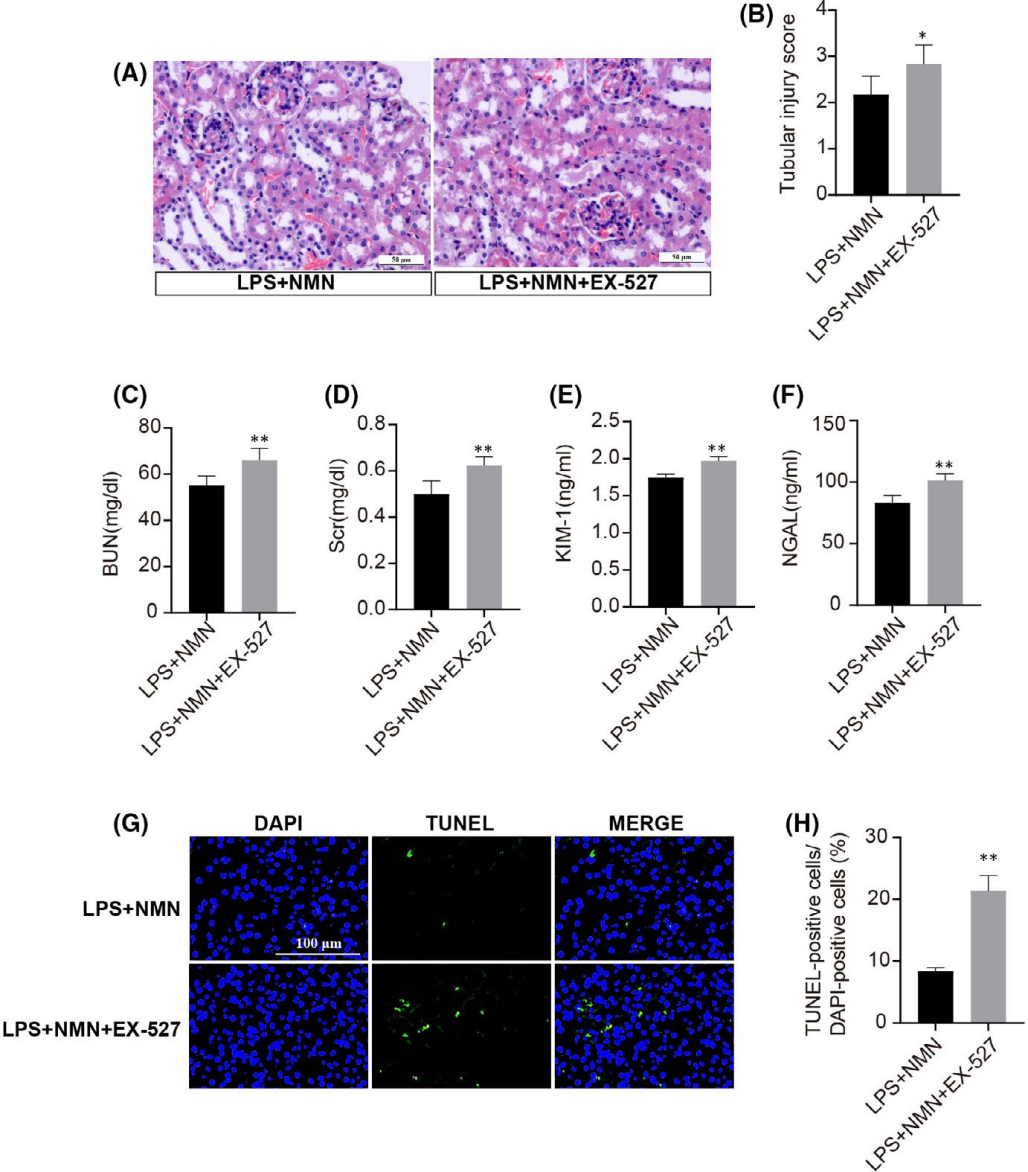
Renoprotective effect of NAD^+^ depends on SIRT1. (A‐B) Representative haematoxylin and eosin‐stained kidney sections (scale bars, 50 μm) and scored damaged tubules in LPS and NMN‐treated C57BL/6 mice with or without EX‐527. Renal functions and damaged markers evaluated by BUN (C), Scr (D), KIM‐1 (E) and NGAL (F) in different groups. (G‐H) Representative TUNEL staining images (scale bars, 100 μm) and quantified by TUNEL‐positive cells in indicated groups. Each result was replicated in three independent experiments, and the values are the mean ±*SD* (*n* = 3). *p < 0.05, **p < 0.01

To explore whether the protective effect of NAD^+^ on sepsis‐induced AKI depends on SIRT1, studies were performed to examine the effect of NMN on SIRT1 inhibitor (EX‐527) under LPS stimulation. As shown in Figure 6, the therapeutic effect of NMN on AKI was substantially diminished by EX‐527, which exhibited significantly higher levels of BUN, Scr, KIM‐1 and NGAL with severe renal damage (Figure 6A‐F). Consistently, the cellular apoptosis assay also supported that the NAD^+^ rescued the LPS‐induced AKI in a SIRT1‐dependent manner and down‐regulation of SIRT1 exacerbated the kidney injury (Figure 6G‐H).

### 3.7 SIRT1 modulates the GSK‐3β/Nrf2 signalling pathway is LPS‐induced AKI

To further investigate the underlying specifically regulation of SIRT1 on sepsis‐induced AKI, we sought to explore molecules that mediate this progress. Glycogen synthase kinase‐3β (GSK‐3β) is known to be ubiquitously expressed in kidneys and as a potential target for relieving LPS‐elicited inflammation and apoptosis.^40^, ^43^ The activity of GSK‐3β is inhibited by phosphorylation at the serine 9 residue and represents as the value of 1‐(p‐GSK‐3β/GSK‐3β).^43^ Burgeoning studies reported that GSK‐3β is crucial for the regulation of NF‐E2‐related factor (Nrf2) and its inactivation up‐regulates the nuclear accumulation of Nrf2 for the antioxidant response.^44^ Given the fact that the anti‐inflammatory and antioxidant properties of SIRT1,^21^ it is conceivable to speculate whether GSK‐3β/Nrf2 is involved in the renoprotective effect of SIRT1 during AKI.

**FIGURE 7:**
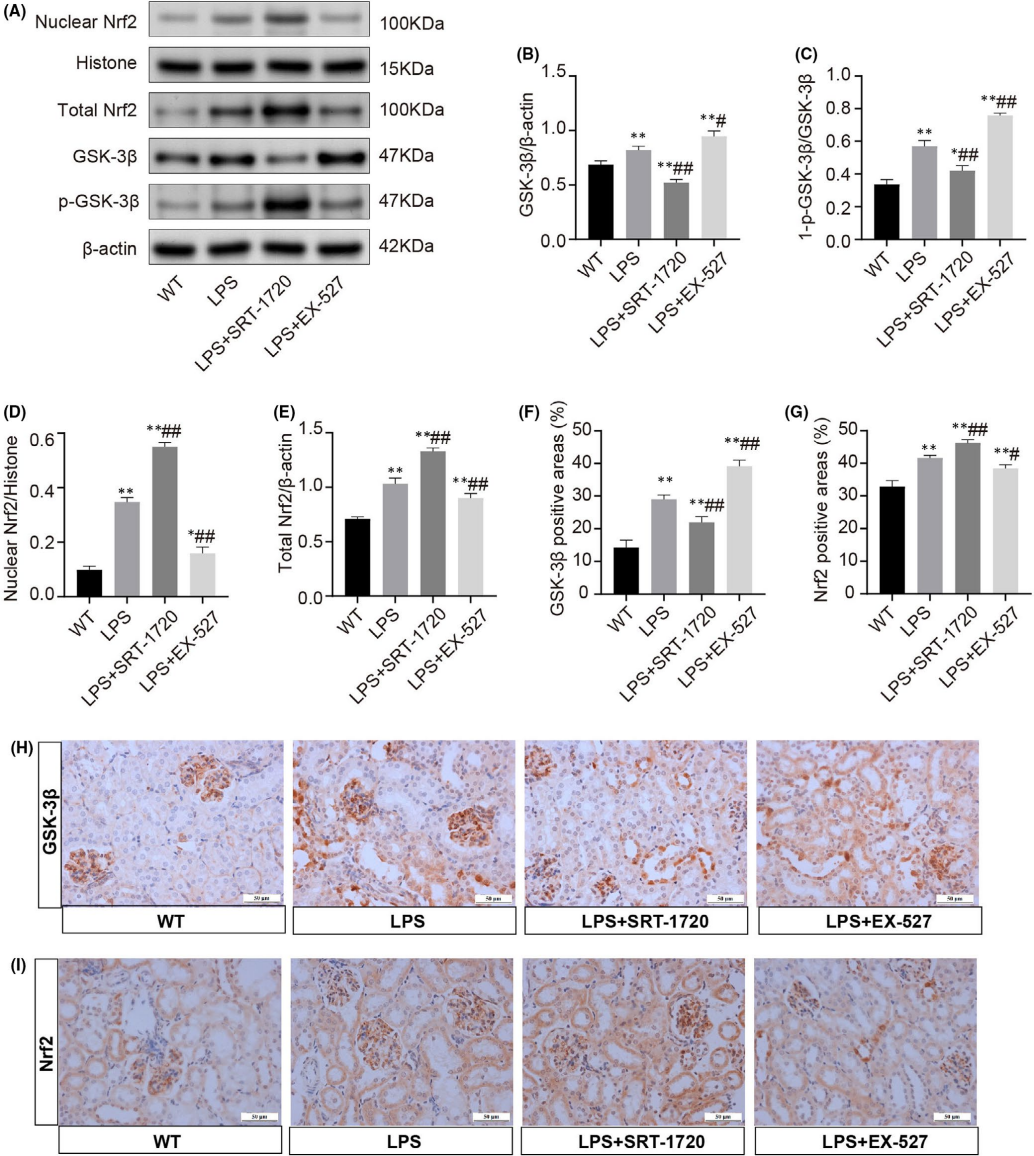
Activation of GSK‐3β/Nrf2 signalling pathway in LPS‐induced AKI. (A) Representative Western blots show the expression of nuclear and total Nrf2, GSK‐3β and p‐ GSK‐3β in the kidney homogenates or nuclear fractions. (B) Relative expression levels of GSK‐3β in the kidney. (C) Values of 1‐ (p‐GSK‐3β/GSK‐3β) in the kidney for each subgroup. (D‐E) Nuclear and total protein expression of Nrf2 in renal tissues. (F‐I) Representative microscopic images of immunohistochemistry staining for GSK‐3β and Nrf2 (scale bars, 50 μm) and followed by quantitative analysis of positive areas. Each result was replicated in three independent experiments, and the data are presented as mean ±*SD* (*n* = 3). *Significance compared with CON group (*p < 0.05, **p < 0.01). #Significance compared with LPS group (^#^ *p *< 0.05, ^##^ *p *< 0.01)

Shown in Figure 7, there were basal levels of GSK‐3β and p‐GSK‐3β under physiologic state, and the expression and activity of GSK‐3β were significantly augmented during LPS stimulation. GSK‐3β levels and activities were significantly lower in LPS+SRT‐1720 mice compared with LPS control, whereas the LPS+EX‐527 group showed apparent elevations. As expected, LPS promoted the expressions of nuclear and total Nrf2 in kidneys, and interestingly, the total and nuclear accumulation of Nrf2 were dramatically increased by SRT‐1720 but diminished by EX‐527. These results indicated that SIRT1 may protect against LPS‐induced AKI in part by regulating the GSK‐3β/Nrf2 signalling pathway.

## 4 DISCUSSION

Previous studies have demonstrated that NAD^+^ precursors, such as nicotinamide mononucleotide (NMN), nicotinamide riboside (NR) and nicotinamide (NAM) attenuate acute renal injury by reducing oxidative stress, inflammation and improving renal functions.^15^, ^24^, ^45^, ^46^ These manners ultimately modulate the concentrations of NAD^+^ and exert the critical role in protecting the kidney from injury.^46^ This study was based on supplementation of NMN or knockdown the key biosynthetic enzyme NMNAT1 to regulate bi‐directionally cellular NAD^+^ levels and identified the NAD^+^‐consuming deacetylase, SIRT1, as an important factor associated with the capacity of NAD^+^ during LPS‐induced AKI. Here, we showed that the AKI had decreased NAD^+^ and SIRT1, and more significantly in NMNAT1^−/−^ mice, whereas the supplementation of NMN restored the contents of NAD^+^ and SIRT1 and rescued LPS‐induced acute renal damage. Activation of SIRT1 greatly facilitated the renal protective effect of NAD^+^ and inhibition of SIRT1 considerably weakened, suggesting that the protective action of NAD^+^ relied on SIRT1. Thus, our study provided the rationale for the therapeutic target of NAD^+^ for LPS‐induced AKI.

Reduction of NAD^+^ or NAD^+^ biosynthetic impairment has been identified in acute kidney injury,^14^, ^15^, ^46^ although the mechanisms have not been fully elucidated. As we know, there are three major pathways contributing to NAD^+^ synthesis in which salvage pathway accounts for most of the NAD^+^ in mammals.^17^, ^47^ Briefly, NAD^+^ is converted from NMN by three isoforms of NMNATs, and NMNAT1 is ubiquitously expressed with highest enzyme activity.^17^, ^48^ Thus, deficit of NMNAT1 could contribute to the insufficient production of NAD^+^ and administration of NMN effectively enhances total cellular NAD^+^ levels. Studies have revealed that renal tubular cells are abundant with mitochondria which highly rely on the NAD homeostasis and are the primary target organelle in AKI.^29^ Mitochondrial dysfunction is not only a common pathologically consequence but also a pathogenic factor, resulting in a vicious cycle that drives the aggravation of AKI,^49^, ^50^ therefore we provided insight into the role of NAD^+^ in mitochondrial functions. By morphology and function analysis, we found that NAD^+^ depletion induced acceleration of mitochondrial deterioration and accumulation of iNOS and ROS, and restoring NAD^+^ levels improved the mitochondrial morphogenesis and functions and decreased the oxidative damage. Further research demonstrated that NAD^+^ homeostasis contributed to protecting against oxidative stress‐induced renal tubular cell apoptosis. Therefore, NAD^+^ might be a specific target in antagonizing LPS‐induced mitochondrial dysfunction and renal tubular cell apoptosis.

SIRT1, an NAD^+^‐dependent deacetylase, has been previously documented as a renal protective factor and therapeutic target for ameliorating of multiple inflammatory diseases.^23^, ^51^, ^52^ Under NMN and NMNAT1^−/−^ conditions, the tendency of SIRT1 was shown consistently with the changes of NAD^+^ levels. Importantly, activation of SIRT1 potently inhibited acute renal tubular damage and apoptosis, while its inactivation markedly aggravated the kidney injury. We further verified whether the regulation of NAD^+^ on sepsis‐related AKI depends on SIRT1. Here, we reported that the protective actions were counterbalanced when restoring NAD^+^ by NMN supplementation in AKI concomitant with inhibition of SIRT1, indicating that the protective effect of NAD^+^ was performed in a SIRT1‐dependent fashion.

GSK‐3β is highly expressed in kidneys and is critical for modulating the self‐defensive response after oxidative stress by promoting Nrf2 nuclear exit and degradation, thus regulating the Nrf2‐mediated antioxidant response.^44^, ^53^, ^54^, ^55^ As a key redox‐sensitive player in oxidative stress, GSK‐3β kinase activity may be amplified by inhibiting phosphorylation at serine 9, which promotes GSK‐3β accumulation and impairs Nrf2‐mediated antioxidant response, driving the exacerbation of oxidative injury.^56^, ^57^ Considering the fact that SIRT1 signalling has been associated with cellular antioxidant defense,^21^ we hypothesized that the SIRT1 protective effect is mediated by GSK‐3β/Nrf2. In agreement, this study showed that GSK‐3β was observably overactive following LPS‐induced AKI, concomitant with a self‐defensive increase of the Nrf2 nuclear accumulation. The activity of GSK‐3β was inhibited by SRT‐1720, the agonist of SIRT1, and the nuclear accumulation of Nrf2 was apparently increased, while EX‐527 exerts the opposite effects. These results indicated that GSK‐3β/Nrf2 may be involved in the process of SIRT1 attenuating AKI.

Although we examined that NAD^+^ levels were negatively correlated with the severity of AKI and the protective effect of NAD^+^ relied on SIRT1, but not yet been validated clinically. Therefore, the effectiveness and mechanisms of NAD^+^ in protecting against AKI remain to be further discovered by a clinical trial in the future.

In conclusion, the study demonstrated that LPS‐induced renal damage is negatively associated with NAD^+^ contents and the expression of SIRT1, and restoring NAD^+^ markedly attenuated AKI possibly by ameliorating mitochondrial dysfunction and apoptosis. We also identified that the renoprotective effects of NAD^+^ are exerted in a SIRT1‐dependent manner by regulation of the GSK‐3β/Nrf2 pathways. This study theoretically provides evidence for the protective effect of NAD^+^ in sepsis‐associated AKI, which may offer a viable approach for AKI therapy.

## CONFLICT OF INTEREST

The authors declare no competing interests.

## AUTHOR CONTRIBUTIONS

**Simeng He:** Methodology (equal); Writing – review & editing (equal). **Qiaoying Gao:** Funding acquisition (equal); Methodology (equal). **Xiaoyang Wu:** Investigation (equal); Methodology (equal). **Jia Shi:** Formal analysis (equal). **Yuan Zhang:** Validation (equal). **Jing Yang:** Methodology (equal). **Xiangyun Li:** Methodology (equal). **Shihan Du:** Methodology (equal); Software (equal). **Yanfang Zhang:** Methodology (equal). **Jian‐Bo Yu:** Funding acquisition (equal); Project administration (equal); Supervision (equal).

## Data Availability

The data that support the findings of this study are available from the corresponding author upon reasonable request.
